# Retraction: Long non-coding RNA XIST promotes cell growth and metastasis through regulating miR-139-5p mediated Wnt/β-catenin signaling pathway in bladder cancer

**DOI:** 10.18632/oncotarget.28314

**Published:** 2022-11-17

**Authors:** Yangyang Hu, Chao Deng, He Zhang, Jing Zhang, Bo Peng, Chuanyi Hu

**Affiliations:** ^1^Department of Urology, Gongli Hospital, The Second Military Medical University, Shanghai 200135, China; ^2^Department of First Clinical Medical College, Nanjing Medical University, Nanjing 211166, China

**This article has been retracted:** Oncotarget requested the original data for this paper to address concerns regarding image duplication. The authors were unable to retrieve the data. As a result, all authors have agreed to the retraction of this paper from Oncotarget.

Original article: Oncotarget. 2017; 8:94554–94568. 94554-94568. https://doi.org/10.18632/oncotarget.21791

